# Application of CD25 and CTLA4 gene transcription levels in early prediction of acute graft-versus-host disease

**DOI:** 10.3389/fimmu.2024.1410439

**Published:** 2024-07-12

**Authors:** Ken Huang, Mengxin Yang, Yuhang Zhou, Yaxuan Cao, Guanxiu Pang, Jie Zhao, Yang Liu, Jianming Luo

**Affiliations:** ^1^ Department of Pediatrics, The First Affiliated Hospital of Guangxi Medical University, Nanning, China; ^2^ Department of Pediatrics, Affiliated Hospital of Youjiang Medical University for Nationalities, Baise, China; ^3^ Institute of Translational Medicine, Hengyang Medical School, University of South China, Hengyang, Hunan, China

**Keywords:** acute graft-versus-host disease, T lymphocyte activation, allogeneic hematopoietic stem cell transplantation, early prediction, biomarker

## Abstract

**Introduction:**

Our study investigated the potential of peripheral blood T cell CD25, CD28, and CTLA-4 gene transcription levels as predictive biomarkers for acute graft-versus-host disease (aGVHD) following allogeneic hematopoietic stem cell transplantation (allo-HSCT).

**Methods:**

Real-time reverse transcription fluorescent quantitative PCR (RT-qPCR) analysis was conducted on day +7, +14, and +21 post-transplantation in patients undergoing allo-HSCT.

**Results:**

Elevated levels of CD25 and CTLA-4 mRNA were found to be associated with the occurrence of aGVHD, as well as severe and gastrointestinal aGVHD. Receiver operating characteristic (ROC) curve analysis was utilized to assess the predictive value of each biomarker. Combined analysis of CD25 and CTLA-4 mRNA levels demonstrated promising predictive potential for aGVHD.

**Conclusion:**

Our results confirmed that the transcription levels of CD25 and CTLA-4 genes could be used as early predictive biomarkers for aGVHD post-allo-HSCT.

## 1 Introduction

Allogeneic hematopoietic stem cell transplantation (allo-HSCT) is a standard validated therapy for patients suffering from malignant and nonmalignant hematological diseases (1). Despite advances in supportive care and transplantation technology, graft-versus-host disease (GVHD) remains a major cause of transplantation-related mortality and morbidity, affecting up to 40–60% of allo-HSCT patients, and accounting for 15–20% of deaths (2).

GVHD is traditionally divided into an acute and chronic disease. Acute GVHD (aGVHD) typically appears in the first 100 days post-transplantation, but can also develop later (3). The incidence of aGVHD is ranges from 30% to 50% (4). In patients transplanted during the periods 1990–1995 and 2011–2015, the incidence of grade II–IV aGVHD was 40% and 28%, respectively, while the incidence of grade III–IV aGVHD was 19% and 11% (5, 6). Recent data from China showed an incidence of moderate and severe acute GVHD ranging from 13% to 47% (7).

Although the overall survival (OS) of patients with aGVHD has been improved over time, the mortality rate remains high (8, 9). One-year OS was 70% in patients with grade II aGVHD and 40% in patients with grade III-IV aGVHD (8). aGVHD severely affects the quality of survival and prognosis of transplant patients. Therefore, early prediction, early prevention and early intervention are the key to reduce the incidence and the severity of aGVHD.

Currently, the diagnosis of aGVHD is mainly based on clinical symptoms, biochemical examinations, imaging or pathological biopsies of the involved organs, but it often lacks specificity and is untimely and invasive, which seriously affects the early diagnosis and treatment of aGVHD. Due to poor reproducibility, there are currently no reliable biomarkers for aGVHD that can be widely used in the clinic practice (10). Therefore, finding early, convenient, and highly specific biological indicators that can predict the occurrence, development and prognosis is of great significance for the prevention and treatment of aGVHD.

GVHD occurs when donor T cells activate and respond to HLA differences on recipient’s tissue (11). The pathophysiology of aGVHD occurs in three phases: initiation phase, T cell activation and the effector phase (5, 12–16). In phase 1, due to the damage of host cells and tissues caused by drugs and (or) whole body irradiation in the pretreatment stage, a large number of endogenous and exogenous molecules are released, which releases a large number of endogenous and exogenous molecules, thus enhancing the presentation of allogeneic antigens by host antigen presenting cells (APCs) and activating APCs. In phase 2, activated host APCs and a large number of cytokines in the early post-transplantation period work together to activate donor T lymphocytes, which proliferate and differentiate into effector cells and secrete cytokines. In phase 3, the activated donor effector T cells and cytokines work together on the target organ, resulting in tissue and organ damage and clinical symptoms of aGVHD.

T lymphocyte activation is a key part of the immune response in aGVHD. Multiple signals are required in the second phase of the GVHD process to enable full-donor T-cell activation and the acquisition of effector function (16). Signal 1 to the T cell involves the ligation of the T-cell receptor (TCR) by major histocompatibility complex (MHC)–peptide complex on the recipient APCs (16). Signal 2 involves costimulation delivered by interaction of costimulatory molecules on the recipient APC surface to their cognate ligand expressed by the donor T cell (16). A third signal is mediated by cytokines secreted by APCs and T cells that bind their relevant receptor on donor T cells to induce proliferation and differentiation into effector T cells (16).

CD28 is the most important co-stimulatory molecules during T cell activation. It is one of the proteins expressed on T cells.CD28 binds to the B7(CD80/86) molecule on APCs and promotes T cell survival, proliferation, and production of a variety of cytokines (16). Cytotoxic T-lymphocyte associated protein 4 (CTLA-4) also known as CD152, is a protein receptor that functions as an immune checkpoint and downregulates immune responses. CTLA4 competitively binds to CD28 and ligand B7(CD80/86) (17). CTLA4 has higher affinity to B7 (CD80/86) ligands, which effectively inhibits the activation of CD28 on T cells and exerts its immunosuppressive effect on T cells (18). The mechanism of CD28 and CTLA4 in aGVHD is complex, and there is a lack of in-depth discussion in the literature at present.

CD25 is one of the markers of T-cell activation. When T cells activated, the expression of CD25 on T cells increases. Several studies have confirmed that CD25 in the serum of patients with aGVHD increases (19–25). CD25 plays an important role in promoting T cell activation, proliferation, and differentiation into effector T cells.

Theoretically, changes in protein levels appear after changes in gene transcription levels. The earlier the disease can be predicted and treated, the more it can reduce the damage to patients. There are no reports of gene transcription levels as early predicting biomarkers for aGVHD. This study attempts to analyze the relationship between the expression of CD28, CTLA4, CD25 on T cells and aGVHD, and to explore the possibility of using the transcriptional levels of CD28, CTLA4 and CD25 to early predict the occurrence of aGVHD and identify severe aGVHD and gastrointestinal aGVHD.

## 2 Materials and methods

### 2.1 Patients

A total of 80 patients who received allo-HSCT in the stem cell transplantation center of pediatrics and hematology department of the First Affiliated Hospital of Guangxi Medical University from September 1, 2021 to November 30, 2022 were selected, including 30 patients with aGVHD as the experimental group and 50 patients without aGVHD as the control group. Diagnosis and grading of aGVHD were based on the clinical and pathological features of the patient, in accordance with the International Federation of Acute Graft- vs.-Host Disease (the Mount Sinai Acute GVHD International Consortium, MAGIC) standards (26). The inclusion criteria were as follows: (1) the patients received allo-HSCT for the first time; (2) the clinical data of the patients were complete and available for analysis; (3) The patients were clinically diagnosed as aGVHD with clear grading. The exclusion criteria were as follows: (1) All cases had severe organ failure occurred within 100 days post- transplantation, including severe pulmonary infection, septicemia, organ failure and organ bleeding; (2) There was still no immune system remodeling within 30 days post-transplantation (neutrophil ≥0.5×10^9^/L for 3 days was granulocyte implantation, platelet count ≥20×10^9^/L for 7 days, and no platelet transfusion was platelet transplant within 7 days) or transplantation failure; (3) loss of visit within 100 days post-transplantation.

### 2.2 Transplant scheme

#### 2.2.1 Mobilization and collection of hematopoietic stem cells

All the HLA 10 loci of unrelated transplantation were identical or only one site was mismatched. All donors were subcutaneously injected with granulocyte colony-stimulating factor (G-CSF) 7.5~10ug/kg.d for hematopoietic stem cell mobilization. After 3–4 days of continuous use, donor peripheral blood stem cells were collected by blood cell separator (COBE Spectra, Unite States). After hematopoietic stem cells were collected, mononuclear cells (MNC) andCD34+ cells were counted by flow cytometry. If the white blood cells (WBC) were more than 50×10^9^/L after mobilization, appropriate reduction of G-CSF should be used.

#### 2.2.2 Conditioning regimen

The preconditioning regimen was selected mainly according to the primary disease, transplant type and HLA compatibility degree. Leukemia, myelodysplastic syndrome (MDS) and thalassemia were included. The regimens of leukemia and MDS include Busulfan (BU) + Cyclophosphamide (CY) +Methylcyclonitrosourea (MeCCNU) + Antithymoglobulin (ATG)/rabbit antilymphocyte globulin produced by Fresenius (ATG-F), BU+CY+ Melphalan (MEL) and Bu+Cy+ Idarubicin (IDA). The details of BU+ CY+ MeCCNU+ATG/ATG-F were as follows: (1)Bu(12.8mg/kg),0.8mg/kg 4 times daily on day -8 to-5; (2) Cy(3.6g/m^2^), 1.8g/m^2^ once daily on day -4 to -3; (3) MeCCNU (250mg/m^2^), oral on day -2; (4) ATG(8mg/kg),2mg/kg once daily on day -4 to-1 or ATG-F (30mg/kg), 7.5mg/kg once daily on day -4 to -1. The details of BU+CY+MEL were as follows: (1) Bu(12.8mg/kg),0.8mg/kg 4 times daily on day-8 to-5; (2) Cy(120mg/kg), 60mg/kg once daily on day -4 to -3; (3) Mel (100mg/m^2^), on day -2. The details of Bu+Cy+IDA were as follows: (1) Bu (12.8mg/kg), 0.8mg/kg 4 times daily on day-7 to -4; (2) Cy (120mg/kg), 60mg/kg once daily on day -3 to-2; (3) IDA (40mg/m2), 20mg/m^2^ once daily on day -3 to -2. The patients with thalassemia were treated with an enhanced regimen Bu+ fludarabine (Flu)+Cy+ATG, and the details were as follows: (1)Bu (16mg/kg), 1mg/kg 4 times daily on day-9 to -6;(2)Flu (150mg/m^2^), 50mg/m^2^ once daily on day -12 to -10; (3)Cy (200mg/kg), 50mg/kg once daily on day -5 to-2; (4) ATG (10mg/kg), 2.5mg/kg once daily on day -5 to -2. (5) All patients were given hydroxyurea 20 mg/kg once daily orally for 2–3 months prior to transplantation.

#### 2.2.3 GVHD prophylaxis

All patients whose donor were related match received a standard immunosuppressive GVHD prophylaxis regimen consisting of Cyclosporine (if HLA matched sibling donor transplantation) or tacrolimus (if related mismatched or unrelated donor transplantation), mycophenolate mofetil (MMF), and short-term methotrexate. Cyclosporine (intravenous, IV) was initiated on day –1 at a dose of 3 mg/kg/day, blood cyclosporine trough level was done twice weekly to maintain the level between 150 and 250 ng/ml. When the patient began to tolerate oral feeding, cyclosporine was shifted to oral route. Tacrolimus (TAC) (IV) was used 1 day prior to transplantation. The initial dosage of TAC was 0.015 mg/kg, twice daily, with intravenous infusion administered over a period of 2 h. Subsequent dosages were adjusted based on the patients’ condition and the plasma concentrations achieved. For patients tolerating oral administration, intravenous TAC was switched to oral TAC. MMF (1.0g/d for adults or children weighing 35≥kg, 30mg/kg/d for children weighing 35<kg) was administered orally in 2 divided doses from day -1 post-transplantation. For sibling compatible transplantation, MMF was discontinued after neutrophil engraftment or day +30. For unrelated donor and haploid transplantation, MMF were halved after neutrophil engraftment and discontinued 2–3 months post-transplantation. Methotrexate (IV) was given on day + 1 with a dose of 15 mg/m2, then 10 mg/m^2^ was given on day + 3, + 6 and + 11(day +11 only for sibling compatible transplantation). Rescue folic acid (IV) at a dose of 15 mg/kg was given 24 h following each dose of methotrexate. In addition, some patients with thalassemia were given CD25 monoclonal antibodies or post-transplant cyclophosphamide (PT-Cy) to strengthen the prevention of GVHD post-transplantation.

### 2.3 Extraction of RNA and DNA

4 mL of early morning peripheral venous blood was collected from patients on day +7, +14, and +21 post-transplantation into EDTA anticoagulated blood collection tubes, and then was placed at 4°C in the refrigerator (The RNA was extracted within 6 hours after the blood was drawn). After the peripheral blood mononuclear cells (PBMCs) were isolated by density gradient centrifugation, RNA was extracted using the Blood Total RNA Kit (Simgen, China), and then the concentration and purity of RNA were detected.The total RNA amount was controlled to 1ug based on the RNA concentration.If the RNA concentration on day +7 does not meet the requirements, an additional 2ml sample will be supplemented the next day for RNA extraction. Qualified RNA was reverse transcribed into cDNA using HiScript III RT SuperMix for qPCR (+gDNA wiper) (Vazyme, China), and all cDNA samples were stored in the refrigerator at -80°C.

### 2.4 RT- qPCR to detect the expression level of CD25, CD28 and CTLA4

The cDNA obtained by reverse transcription was amplified by 7500 real-time fluorescence quantitative PCR instrument (Applied Biosystems, Unite States) and ChamQ Universal SYBR qPCR Master Mix (Vazyme, China). Details of primers are shown in **Table 1**, in which GAPDH is the internal reference. The total volume of the reaction system was 20 μ L, including 10μL of 2×ChamQ Universal SYBR qPCR Master Mix, 0.4μL of each forward and reverse primers, 7.2 μL of ddH2O, and 2 μL of cDNA, respectively. PCR reaction conditions: preheating at 95°C for 30 seconds; Denaturing at 95°C for 10 seconds, annealing at 60°C for 20 seconds, a total of 40 cycles. The relative expression levels of CD28, CD25 and CTLA-4 mRNA were analyzed by the 2^-ΔΔCT^ method using GADPH as the internal reference. The RT-qPCR products were detected by electrophoresis.

**Table 1:** Primer sequence, length, product length.

Gene	Direction of primers	Primer sequence (5’→3’)	Primer length	Tm	CG%	Product length
CD28	Forward primer	GCCCATCGTCAGGACAAAGA	20	60.04	55.00	154
	Reverse primer	TGGACAAAGGTGTTT CCAGCTA	22	59.83	45.45	
CD25	Forward primer	ATCAGTGCGTCCAGGGATAC	20	59.25	55.00	150
	Reverse primer	GAGGCTTCTCTTCACCT GGAA	21	59.37	52.38	
CTLA4	Forward primer	CCCTGTCTTCTGCAAAGCAAT	21	59.11	47.62	166
	Reverse primer	CGCACAGACTTCAGTCACCT	20	59.97	55.00	
GAPDH	Forward primer	CAGGAGGCATTGCTGATGAT	20	58.02	50.00	138
	Reverse primer	GAAGGCTGGGGCTCATTT	18	57.23	55.56	

### 2.5 Statistical analysis

Statistical analysis was performed using the statistical analysis software SPSS (version 26.0), and the application software Graph Pad Prism (version 9.0) was used to draw graphs. The counting data were expressed by the number of cases and percentage, and the chi-square test was used for comparison between groups. The measurement data conforming to normal distribution were expressed as mean ± standard deviation (X ± S), and the comparison between groups was conducted by independent sample t test or paired sample T test (paired sample). Data that did not conform to normal distribution were represented by median and interquartile interval M (P25, P75), and Mann-Whitney U test or Wilcoxon Signed Rank Test (paired sample) was used for comparison between groups. If the measurement data conformed to the normal distribution, the correlation analysis between the two groups was analyzed using the Pearson method, and if the measurement data did not conform to the normal distribution, the Spearman method was used. The joint prediction probability was generated by binary Logistic regression. ROC and area under the curve (AUC) were used to analyze the early predicting value of CD25, CD28 and CTLA-4 mRNA to aGVHD. A two-sided P<0.05 was considered statistically significant.

## 3 Results

### 3.1 General information

The clinical characteristics and transplant status of 80 patients were listed in **Tables 2**, **3**. In our study, a total of 80 patients who receive allo-HSCT were included, comprising 49 males and 31 females. The median age was 10.79 (6.21~16.12), the maximum age was 50.46 years old, and the minimum age was 2.15 years old. There were 61 cases of thalassemia (55 cases of β-thalassemia major and 6 cases of hemoglobin H disease), 10 cases of acute lymphoblastic leukemia (ALL), 7 cases of acute myeloid leukemia (AML), 1 case of chronic myeloid leukemia (CML) and 1 case of myelodysplastic syndrome (MDS). Among the transplantation types, there were 14(17.5%) cases of related complete HLA matching, 40(50.0%) cases of related incomplete HLA matching, 22 (27.5%) cases of non-related HLA matching, and 4 (5.00%) cases of non-related incomplete HLA matching. The stem cell sources were bone marrow, peripheral blood, bone marrow + peripheral blood, peripheral blood + umbilical cord blood and bone marrow + umbilical cord blood, and the percentages of them were 12.5% (n=10), 35.0%(n=28), 46.3%(n=37), 2.5% (n=2) and 3.8% (n=3) respectively. All patients were successfully implanted. The median time of granulocyte engraftment was 12.0(11.0, 14.0) days. There were 5 cases with platelet level not lower than 20×10^9^/L post-transplantation, including 4 cases in control group and 1 case in experimental group, and the median time of platelet engraftment in 75 patients was 14.0(12.0, 17.0) days. The median input of monocytes and CD34+ cells were 10.11(8.18,12.03) ×10^8^/kg and 7.64(6.49,8.98) ×10^6^/kg respectively. A total of 30 patients with aGVHD were included in the experimental group. During the perioperative period and 30 days post-transplantation (day-12 to +30), 7(23.3%) cases had bacterial infection, 11(36.7%) cases had viral infection, 8(26.7%) cases had bacterial + viral infection, and 1(3.3%) case had bacterial + viral + fungal infection. A total of 50 patients without aGVHD were included in the control group. During the perioperative period and 30d post-transplantation, 9(18.0%) cases had bacterial infection, 16(32.0%) cases had viral infection, 6(12.0%) cases had bacterial + viral infection, and 3(6.0%) cases had bacterial + viral + fungal infection. Except for blood type of donor-recipient, there were no statistically significant differences in age, sex of donor, Donor–recipient gender match, ABO mismatch, donor type, underlying disease, stem cell source, conditioning regimen, GVHD prophylaxis, engraftment times of neutrophil and platelet, input of monocytes and CD34+ cells, and Infection peri- and post-HSCT between experimental group and control group (P≥0.05).

**Table 2:** Clinical characteristics and transplant of patients.

Variables	Total	Non- aGVHD	aGVHD	P
	(n=80)	(n=50)	(n=30)	
Age at HSCT, median (IQR)	10.79(6.21,16.12)	10.93(5.80,16.35)	10.20(6.39,13.23)	0.788
Sex, n (%)				
Male	49(61.3)	31(62.0)	18(60.0)	0.859
Female	31(38.8)	19(38.0)	12(40.0)	
Donor–recipient gender match, n (%)				
Male to male	30(37.5)	19(38.0)	11(36.7)	0.682
Male to female	22(27.5)	12(24.0)	10(33.3)	
Female to female	9(11.3)	7(14.0)	2(6.7)	
Female to male	19(23.8)	12(24.0)	7(23.3)	
ABO mismatch, n (%)				
None	31(38.8)	21(42.0)	10(33.3)	0.045
Major	19(23.8)	9(18.0)	10(33.3)	
Minor	22(27.5)	12(24.0)	10(33.3)	
Bidirectional	8(10.0)	8(16.0)	0(0.0)	
Donor type, n (%)				
Matched related	14(17.5)	11(22.0)	3(10.0)	0.160
Mismatched related	40(50.0)	27(54.0)	13(43.3)	
Matched unrelated	22(27.5)	10(20.0)	12(40.0)	
Mismatched unrelated	4(5.00)	2(4.00)	2(6.70)	
Underlying disease, n (%)				
Thalassaemia	61(76.3)	38(76.0)	23(76.7)	0.314
ALL	10(12.5)	8(16.0)	2(6.7)	
CML/AML	8(10.0)	4(8.0)	4(13.3)	
MDS	1(1.3)	0(0.0)	1(3.3)	
Stem cell source, n (%)				
BM	10(12.5)	6(12.0)	4(13.3)	0.418
PB	28(35.0)	14(28.0)	14(46.7)	
BM+PB	37(46.3)	26(52.0)	11(36.7)	
PB+CB	2(2.5)	2(4.0)	0(0.0)	
BM+CB	3(3.8)	2(4.0)	1(3.3)	
Conditioning regimen, n (%)				
BU+CY+Flu+ATG	61(76.3)	38(76.0)	23(76.7)	0.676
BU+CY+MeCCNU+※	13(16.3)	9(18.0)	4(13.3)	
BU+CY+MEL	5(6.3)	3(6.0)	2(6.70)	
BU+CY+IDA	1(1.3)	0(0.0)	1(3.3)	
GVHD prophylaxis, n(%)				
FK506+MMF+MTX	36(45.0)	20(40.0)	16(53.3)	0.325
FK506+MMF+MTX+△	22(27.5)	14(28.0)	8(26.7)	
Cy+MMF+MTX	16(20.0)	13(26.0)	3(10.0)	
Cy +MMF+MTX+△	3(3.8)	1(2.0)	2(6.7)	
Cy +MMF+MTX+○	3(3.8)	2(4.0)	1(3.3)	
Engraftment				
Neutrophil, median (IQR)	12.0(11.0,14.0)	12.0(11.0,13.3)	12.5(11.0,14.3)	0.256
Platelet, median (IQR)	14.0(12.0,17.0)	13.5(11.0,16.75)	15.0(13.0,17.0)	0.107
Graft, M(IQR)				
MNC(×10^8^/kg)	10.11(8.18,12.03)	9.76(7.80,11.49)	10.18(8.29,13.39)	0.447
CD34+(×10^6^/kg)	7.64(6.49,8.98)	7.90(6.88,9.57)	7.41(6.20,8.48)	0.193
Infections peri- and post-HSCT,n(%)				
None	19(23.8)	16(32.0)	3(10.0)	0.133
Bacterial	16(20.0)	9(18.0)	7(23.3)	
Viral	27(33.8)	16(32.0)	11(36.7)	
Bacterial + viral	14(17.5)	6(12.0)	8(26.7)	
Bacterial + fungal+ viral	4(5.0)	3(6.0)	1(3.3)	

※: ATG/ATG-F, ATG is Antithymocyte globulin (Antithymoglobulin), ATG-F is rabbit antilymphocyte globulin produced by Fresenius.△: anti-CD25 monoclonal antibody; ○: Post-transplant cyclophosphamide, PT-Cy; IQR, Interquartile range; ALL, Acute lymphoblastic leukemia; CML, Chronic myeloid leukemia; AML, Acute myeloid leukemia; MDS, Myelodysplastic syndrome; BU, Busulfan; CY, Cyclophosphamide; Flu, Fludarabine; MeCCNU, methylchloroethylnitrosourea; MEL, Melphalan; IDA, Idarubicin; FK506, Tacrolimus; MMF, Mycophenolate mofetil; MTX, Methotrexate; MNC, Mononuclear cells.

**Table 3:** The manifestations of the patients with aGVHD.

Variables	Value
Total, n	30
Time from stem cell transplantation to aGVHD onset, M(IQR)	28.5(21.8,36.0)
Number of cases with onset after day +7, n (%)	30(100.0)
Number of cases with onset after day +14, n (%)	29(96.7)
Number of cases with onset after day +21, n (%)	25(83.3)
aGVHD Grade, n(%)	
I	12(40.0)
II	7(23.3)
III	4(13.3)
IV	7(23.3)
II-IV	18(60.0)
Affected organs by aGVHD, n(%)	
Skin alone	14(46.7)
Gut alone (a)	9(30.0)
Skin + Gut (b)	7(23.3)
Skin + Liver+ Gut (c)	1(3.3)
Gut (including a+b+c)	16(53.3)

Among the 30 cases of aGVHD, there were 12 cases of grade I (40.0%), 7 cases of grade II (23.3%), 4 cases of grade III (13.3%), 7 cases of grade IV (23.3%) and 18 cases of grade II-IV (60.0%). The organ involvement of aGVHD included 14 (46.7%) cases of skin alone, 9 (30.0%) cases of gut alone, 7 (23.3%) cases of skin + gut, and 1 (3.3%) case of skin+ gut+liver. Gut involvement occurred in 16 (53.3%) of all cases, and all patients with gut involvement underwent colonoscopic biopsy. The median onset time of aGVHD post-transplantation was + 28.5 (21.8) days.30 (100.0%) cases after + 7 days, 29 (96.7%) cases after + 14 days, and 25 (83.3%) cases after + 21 days.

### 3.2 Gene transcription changes in transplant recipients with non-aGVHD and aGVHD

**Figure 1:**
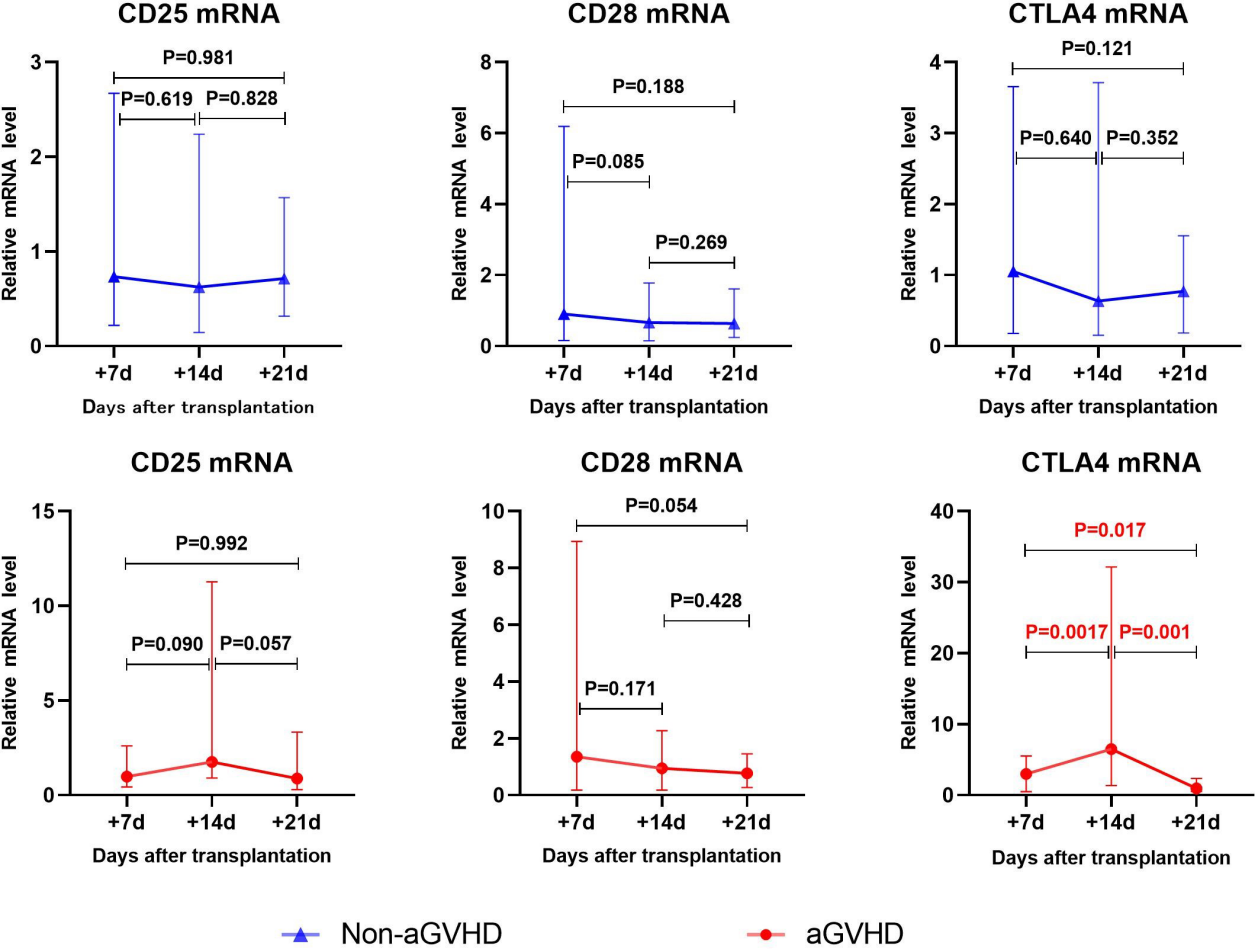
The changes of CD25、CD28、CTLA4 mRNA levels in aGVHD group and non- aGVHD group post- transplantation.

Gene transcription changes post-transplantation was shown in **Figure 1**. In the non-aGVHD group, there were no significant changes in the expression of CD25, CD28 and CTLA4 on T cells. In the aGVHD group, CD25 mRNA showed an upward trend from day +7 to day +14 after transplantation (P=0.09), and a downward trend from day +14 to day +21 post-transplantation (P=0.057). CTLA4 mRNA significantly increased from day +7 to day +14 post-transplantation (P=0.0017), and decreased significantly from day +14 to day +21 post-transplantation (P=0.001). CD28 mRNA showed a downward trend from day +7 to day +21 post-transplantation (P=0.054).

### 3.3 Early prediction of aGVHD occurrence by CD25, CD28 and CTLA4 mRNA

#### 3.3.1 Univariate analysis

Univariate analysis was used to analyze the levels of CD25, CD28 and CTLA4 mRNA in the experimental group and the control group, as shown in **Table 4**. The results showed that between the experimental group(aGVHD group) and the control group(non-aGVHD group) on day +14, the median relative expressions of CTLA4 mRNA were 0.6239 (0.1441, 2.2391) and 1.7585 (0.9012, 11.2691), respectively, and the median relative expressions of CTLA4 mRNA were 0.6359 (0.1502, 3.7119) and 6.2940 (1.0401, 30.0601), respectively. Both levels were higher in the experimental group than in the control group, and the differences were statistically significant (p < 0.05). There were no differences in the levels on day +7 and +21 post-transplantation between the two groups.

**Table 4:** Comparison of gene transcription levels between non-aGVHD group and aGVHD group.

Variables	Days post-transplantation	non-aGVHD group	aGVHD group	P
		(Relative mRNA expression level)	(Relative mRNA expression level)	
CD25	+7d	0.7342(0.2188,2.6719)	0.9758(0.4299,2.6102)	0.340
	+14d	0.6239(0.1441,2.2391)	1.7585(0.9012,11.2691)	0.001
	+21d	0.7140(0.3163,1.5683)	0.8806(0.2895,3.3314)	0.462
CD28	+7d	0.8276(0.1365,5.4581)	1.3581(0.1805,8.9358)	0.561
	+14d	0.4761(0.1183,1.4806)	0.9487(0.1819,2.2689)	0.218
	+21d	0.5512(0.2230,1.8319)	0.7712(0.2713,1.4580)	0.598
CTLA4	+7d	1.0523(0.1795,3.6547)	3.0029(0.5145,6.4521)	0.090
	+14d	0.6359(0.1502,3.7119)	6.2940(1.0401,30.0601)	0.001
	+21d	0.7720(0.1861,1.5535)	0.9132(0.5061,2.3147)	0.241

The red colored values indicate P<0.05, which is statistically significant.

#### 3.3.2 Correlation analysis

**Figure 2:**
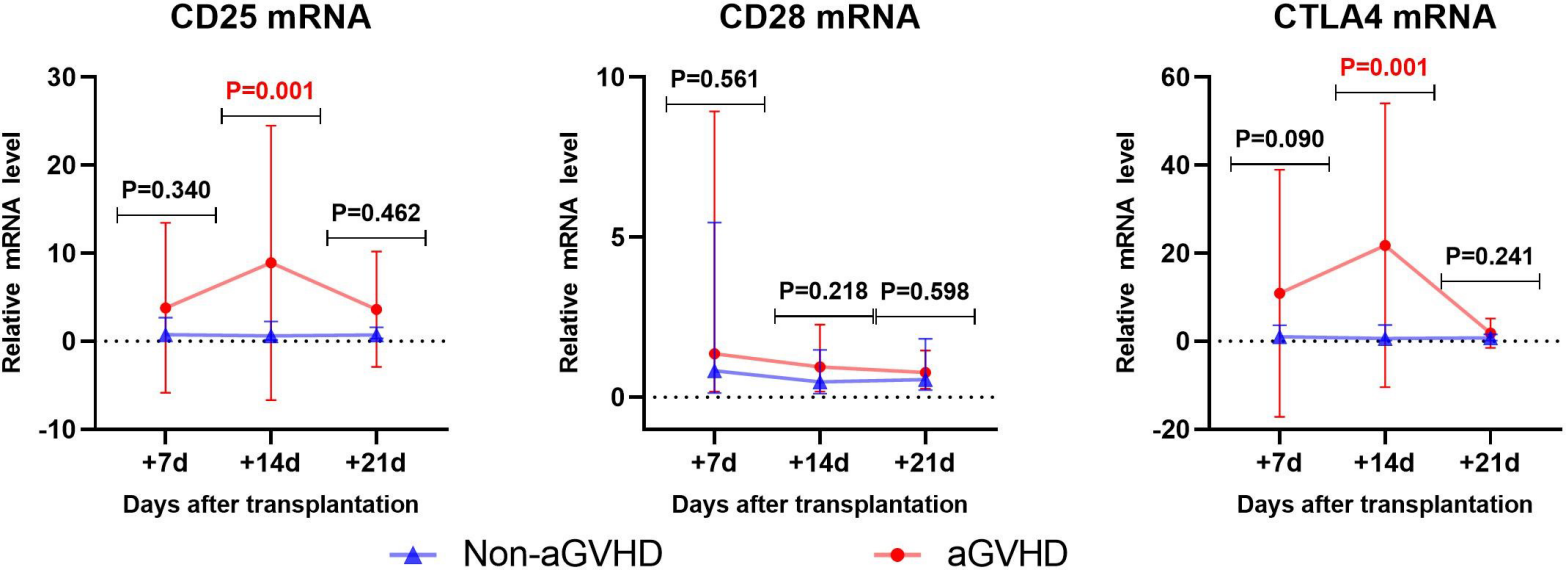
Comparison of gene transcription levels between non-aGVHD group and aGVHD group.

By using Spearman method to analyze the correlation of the transcriptional levels between CD25 and CTLA4 on day+14 post-transplantation, the results showed that the correlation coefficient was 0.365 (P=0.001), as shown in **Figure 2**.

#### 3.3.3 ROC analysis

**Figure 3:**
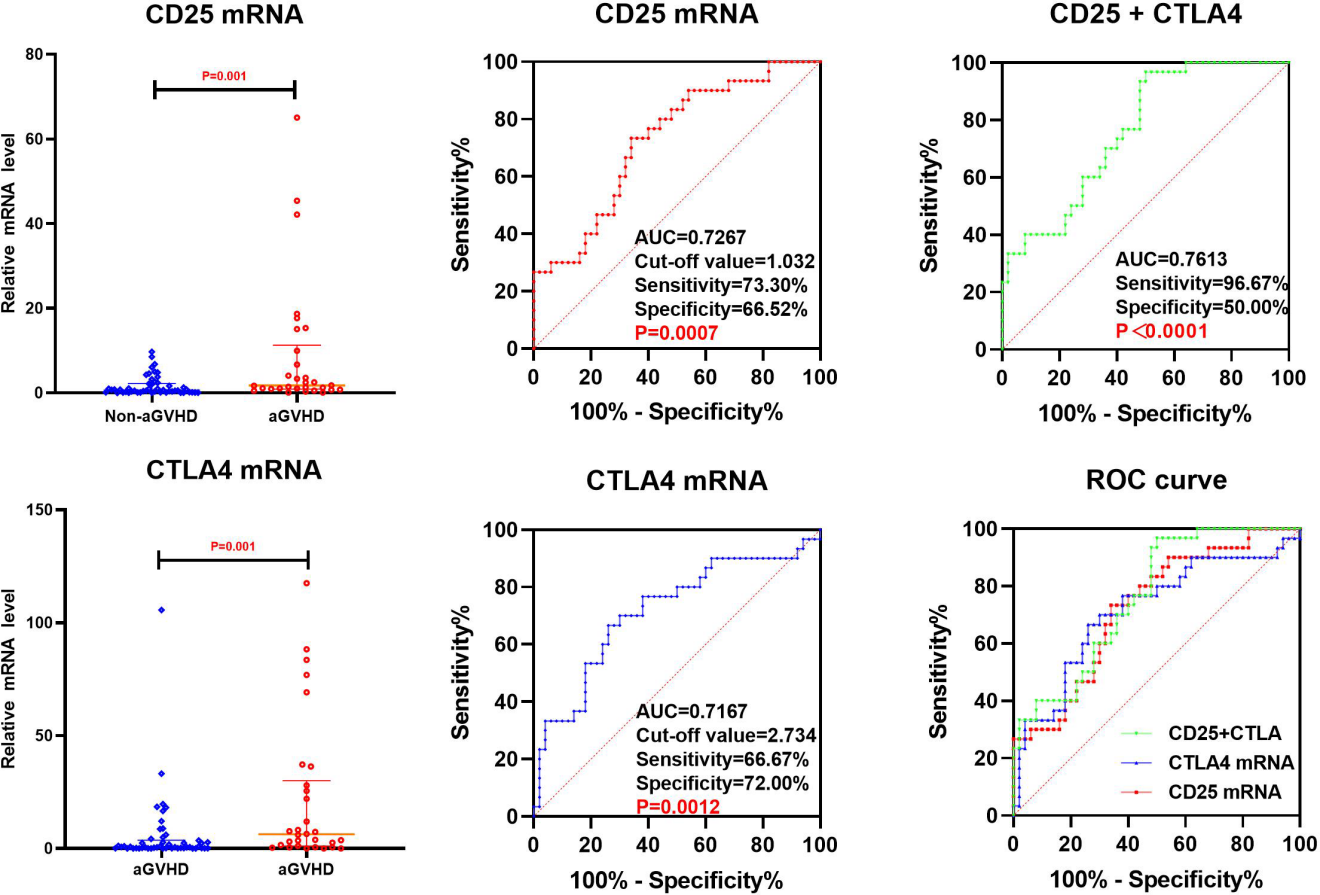
ROC analysis results of CD25 and CTLA4 mRNA to predict the occurrence of aGVHD.

Binary logistic regression was performed on CD25 and CTLA4 mRNA at +14 days post-transplantation, with the method “forward: conditional” selected, and predicted probabilities were calculated and saved. The prediction probability generated by binary Logistic regression and the mRNA of CD25 and CTLA4 on day +14 post-transplantation were analyzed by ROC (**Figure 3**). The results showed that the AUC of CD25 and CTLA4 mRNA on day +14 post-transplantation predicting aGVHD occurrence were 0.7267(73.30%,66.52%) and 0.7167(66.67%,72.00%), and the cut-off values were 1.032 and 2.734, respectively. The AUC of CD25 and CTLA4 mRNA for combined prediction of aGVHD occurrence was 0.7613 (96.67%,50.00%), and the AUC of combined prediction was slightly improved.

### 3.4 Early prediction of Grade II-IV aGVHD by CD25, CD28 and CTLA4 mRNA

#### 3.4.1 Univariate analysis

**Figure 4:**
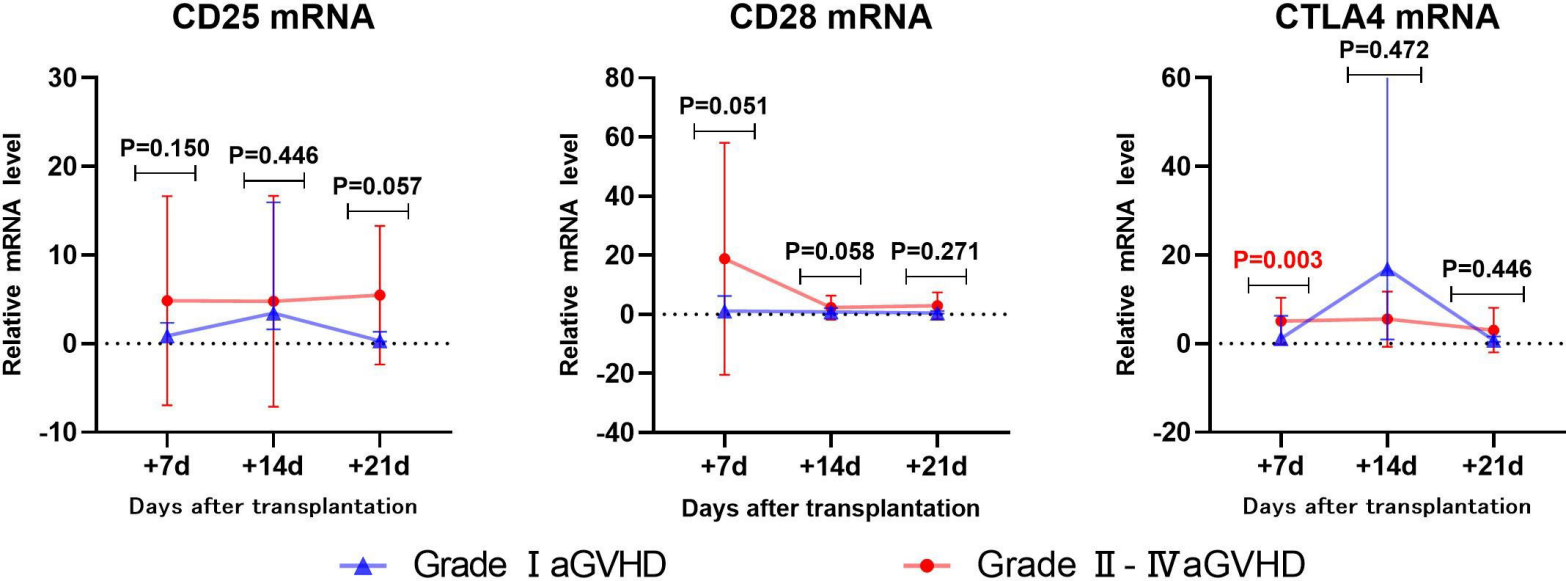
Comparison of mRNA levels of CD25, CD28 and CTLA4 in Grade I aGVHD and Grade II-IV aGVHD.

The transcriptional levels of CD25, CD28, and CTLA4 were assessed by univariate analysis in both grade I and grade II-IV aGVHD groups, as shown in **Table 5** and **Figure 4**. The results revealed that on day +7 post-transplantation, the median relative expression of CTLA4 mRNA was 0.4874 (0.1311, 2.5504) in the grade I aGVHD group and 3.8623 (2.4291, 9.3152) in the grade II-IV aGVHD group. The level in grade II-IV aGVHD group was significantly higher than that in the grade I aGVHD group(P=0.003). There was no difference between the grade I aGVHD group and the grade II-IV aGVHD group on day +14 and +21 post-transplantation. There were no differences in relative expression of CD25 and CD28 mRNA between the two groups on day + 7, + 14, and + 21 post-transplantation.

**Table 5:** Comparison of mRNA levels of CD25, CD28 and CTLA4 in grade I aGVHD and grade II-IV aGVHD.

Variables	Days post- transplantation	grade I aGVHD group	grade II-IV aGVHD group	P
		(Relative mRNA expression level)	(Relative mRNA expression level)	
CD25	+7d	0.7085(0.1950,1.2258)	1.0223(0.5785,3.1855)	0.150
	+14d	3.0428(0.9190,13.8356)	1.3420(0.8854,8.8589)	0.446
	+21d	0.3146(0.1931,1.0828)	1.3458(0.5470,4.7185)	0.057
CD28	+7d	0.2294(0.0440,7.5099)	2.5083(0.3515,14.2958)	0.051
	+14d	1.7611(0.0979,3.4504)	0.8931(0.2233,1.8740)	0.582
	+21d	0.6108(0.2135,1.3709)	0.9084(0.3813,1.6865)	0.271
CTLA4	+7d	0.4874(0.1311,2.5504)	3.8623(2.4291,9.3152)	0.003
	+14d	16.6034(1.7693,36.9906)	4.9941(0.5875,14.4626)	0.472
	+21d	0.8056(0.3316,1.5077)	0.9198(0.5142,2.4935)	0.446

The red colored values indicate P<0.05, which is statistically significant.

#### 3.4.2 ROC analysis

**Figure 5:**
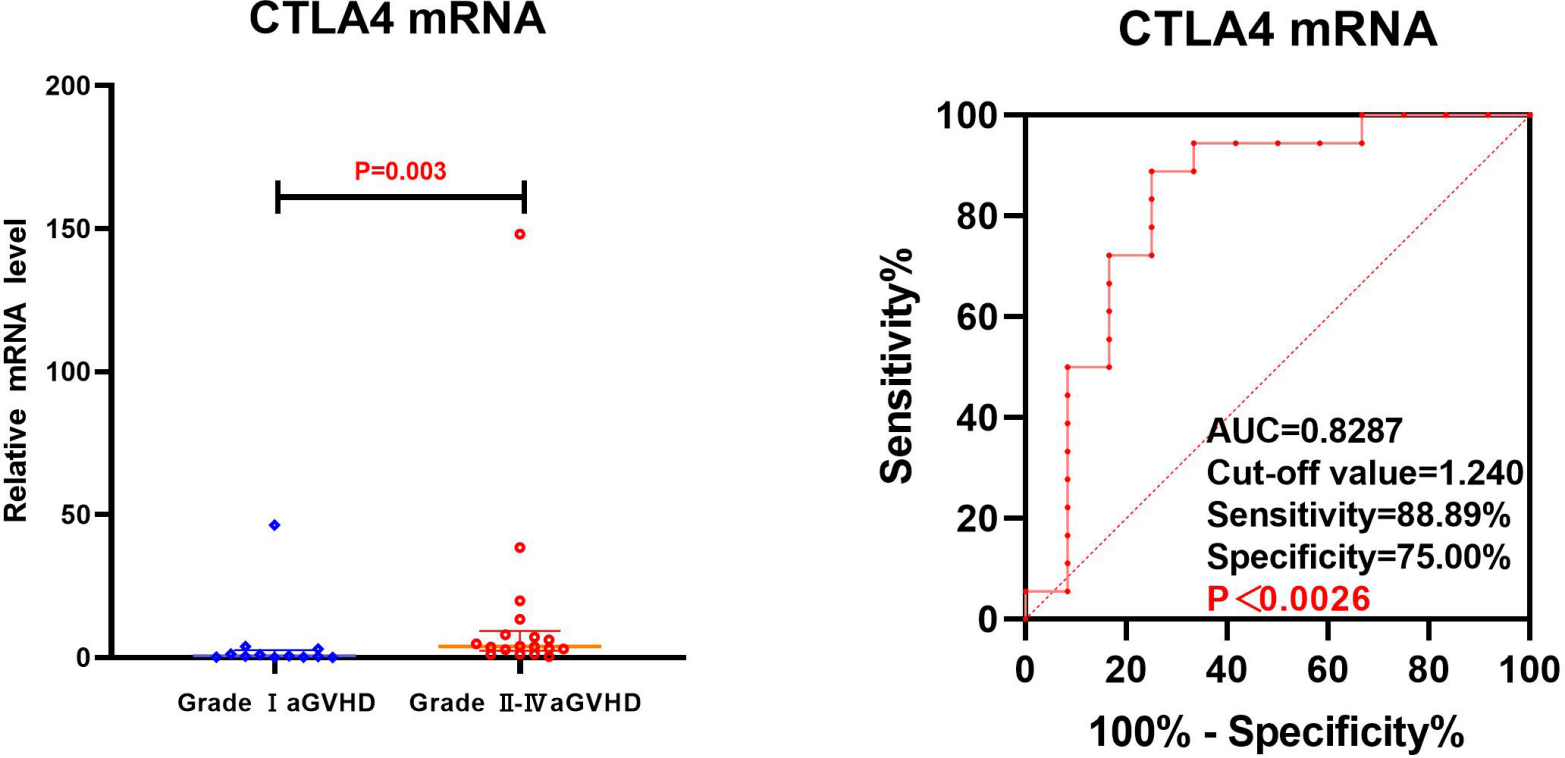
ROC analysis of CTLA4 mRNA to predict Grade II-IV aGVHD on day +7 post-transplantation.

The CTLA4 mRNA on day +7 post-transplantation was analyzed by ROC. The results show that the AUC of CTLA4 mRNA predicting grade II-IV aGVHD was 0.8287 (88.89%,75.00%), with Cut-off value=1.240, as shown in **Figure 5**.

### 3.5 Early prediction of gastrointestinal aGVHD by CD25, CD28 and CTLA4 mRNA

#### 3.5.1 Univariate analysis

**Figure 6:**
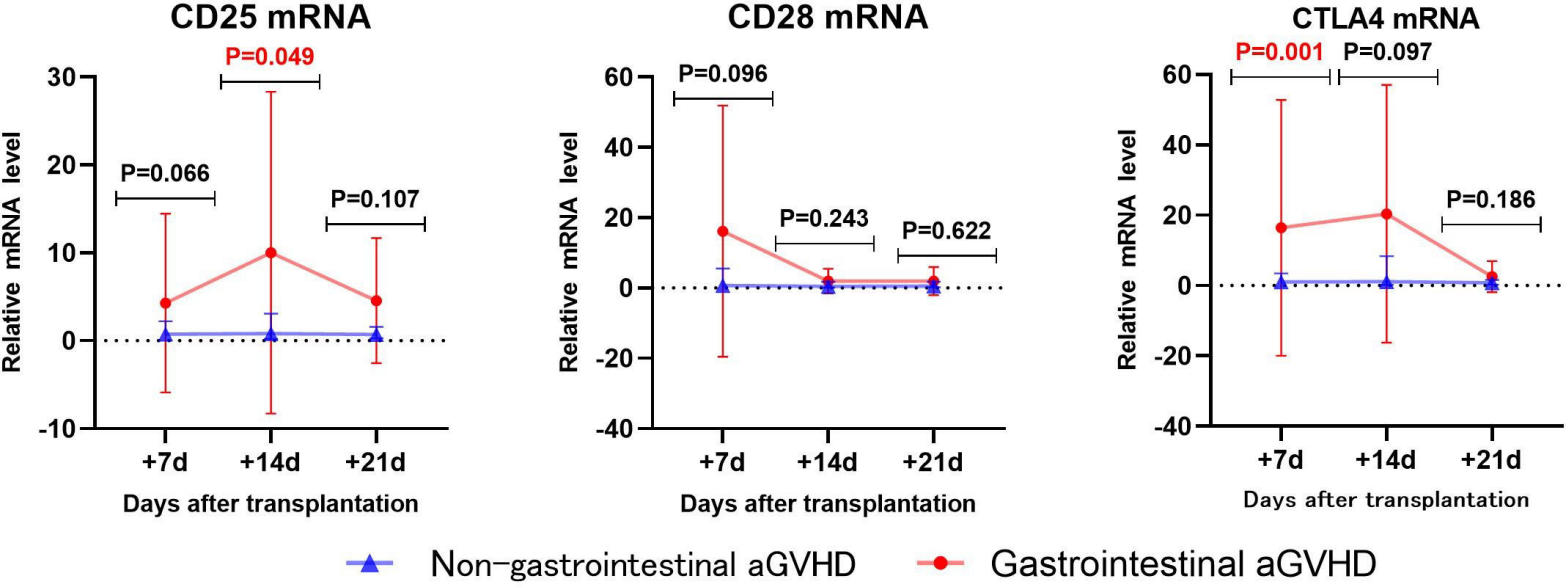
Comparison of mRNA levels of CD25, CD28 and CTLA4 in gastrointestinal and non-gastrointestinal aGVHD.

The levels of CD25, CD28, and CTLA4 mRNA were analyzed using univariate analysis in both the non-gastrointestinal aGVHD and gastrointestinal aGVHD groups, as shown in **Table 6** and **Figure 6**. It was observed that on day +7 post-transplantation, the median relative expression of CTLA4 mRNA was 1.0074 (0.1820, 3.4584) in the gastrointestinal non-aGVHD group and 3.8623 (2.8670, 12.0236) in the gastrointestinal aGVHD group. The level of CTLA4 mRNA was significantly higher in the gastrointestinal aGVHD group compared to the non-gastrointestinal aGVHD group, indicating a statistically significant difference between the two groups. The results also revealed that on day +14 post-transplantation, the median relative expression of CD25 mRNA was 0.8251(0.2229,3.0832) in the non-gastrointestinal aGVHD group and 1.5744(0.8045,13.1897) in the gastrointestinal aGVHD group. The level of CD25 mRNA was significantly higher in the gastrointestinal aGVHD group compared to the non-gastrointestinal aGVHD group, indicating a statistically significant difference between the two groups. There was no difference in the levels of CTLA4 mRNA on day +14 and +21, as well as the levels of CD25 mRNA on day +7 and +21 post-transplantation. There were no differences in the levels of CD28 mRNA on day +7, +14 and +21 post-transplantation between the two groups.

**Table 6:** Comparison of CD25, CD28 and CTLA4 mRNA levels between non-gastrointestinal and gastrointestinal aGVHD.

Variables	Days post-transplantation	non-gastrointestinal aGVHD (n=64)	gastrointestinal aGVHD (n=16)	P
		(Relative mRNA expression level)	(Relative mRNA expression level)	
CD25	+7d	0.7342(0.2466,2.2146)	1.0223(0.6165,3.2885)	0.066
	+14d	0.8251(0.2229,3.0832)	1.5744(0.8045,13.1897)	0.049
	+21d	0.7140(0.2839,1.5743)	1.0754(0.4635,6.6300)	0.107
CD28	+7d	0.7360(0.1308,5.5428)	2.5083(0.4109,12.2925)	0.096
	+14d	0.4761(0.1149,1.7702)	0.9487(0.3275,2.1045)	0.243
	+21d	0.5805(0.2181,1.7619)	0.8040(0.3128,1.3369)	0.622
CTLA4	+7d	1.0074(0.1820,3.4584)	3.8623(2.8670,12.0236)	0.001
	+14d	1.0852(0.1899,8.3905)	4.9941(0.5356,11.0084)	0.097
	+21d	0.7720(0.2527,1.6105)	0.9198(0.5328,2.6463)	0.186

The red colored values indicate P<0.05, which is statistically significant.

#### 3.5.2 ROC analysis

**Figure 7:**
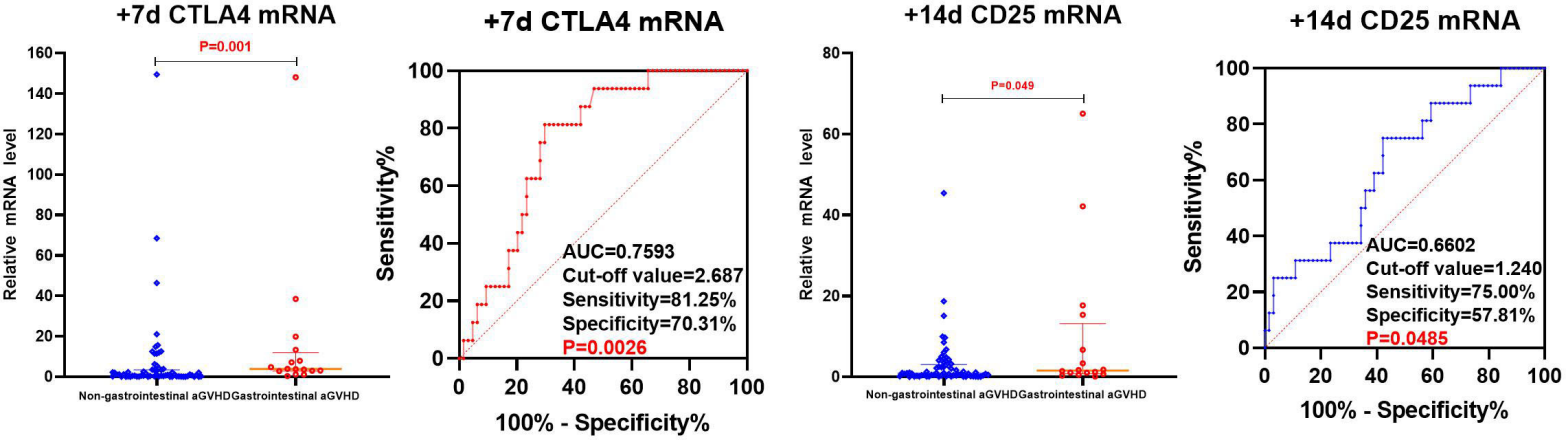
ROC analysis of CD25 and CTLA4 mRNA levels to predict gastrointestinal aGVHD.

The CTLA4 mRNA on day +7 and CD25 mRNA on day +14 post-transplantation predicting gastrointestinal aGVHD were analyzed by ROC, as shown in **Figure 7**. The results showed that the AUC of CTLA4 mRNA on day +7 and CD25 mRNA on day +14 predicting gastrointestinal aGVHD were 0.7593 (81.25%, 70.31%), 0.6602 (75.00%, 57.81%), and the Cut-off value were 2.687 and 1.240, respectively.

## 4 Discussion

aGVHD remains a common post-transplant complication and one of the leading causes of death (27). aGVHD seriously affects the quality of life and prognosis of transplant patients. Early prediction, early prevention and early intervention are the key to reduce the incidence and the severity of aGVHD. Here, we explore the possibility of using the transcriptional levels of CD28, CTLA4 and CD25 genes on T cells to early predict the occurrence of aGVHD and identify severe aGVHD. In addition, once gastrointestinal aGVHD occurs, the disease progresses rapidly, the treatment is difficult, and the mortality rate is high (28, 29). Clinicians attach great importance to gastrointestinal aGVHD. Therefore, we also explored the early prediction for gastrointestinal aGVHD.

Except for blood type of donor-recipient, there were no statistically significant differences in age, sex of donor, donor–recipient gender match, ABO mismatch, donor type, underlying disease, stem cell source, conditioning regimen, GVHD prophylaxis, engraftment times of neutrophil and platelet, input of monocytes and CD34+ stem cells, and Infection peri- and post-HSCT between experimental group and control group. To some extent, these balanced the influence of factors such as infection on the results of the study.

aGVHD is an immune response caused by donor T lymphocytes recognizing recipient histiocyte with different genetics. T lymphocytes play a role in tissue cell injury through activation, proliferation and differentiation into effector cells (30). The activation of T lymphocytes is the key link of immune response. CD28 and CTLA-4 are the most important co-stimulatory molecules during T cell activation (31). CD28 binds to the B7(CD80/86) molecule on APCs and promotes T cell survival, proliferation, and production of a variety of cytokines (16). CTLA-4 is a protein receptor that functions as an immune checkpoint and downregulates immune responses. CTLA4 competitively binds to CD28 and ligand B7(CD80/86) (17). CTLA-4 has higher affinity to B7 (CD80/86) ligands (32), which effectively inhibits the activation of CD28 on T cells and exerts its immunosuppressive effect on T cells (18). In the aGVHD group, CTLA4 mRNA significantly increased from day +7 to day +14 (P=0.017), and decreased significantly from day +14 to day +21 (P=0.001). CD28 mRNA showed a downward trend from day +7 to day +21 (P=0.054). It is worth noting that the expression of CD28 increased earlier, and then decreased, while CTLA4 lagged behind the increase of CD28 expression, and then decreased rapidly. Our results similarly validate the involvement of CD28 and CTLA-4 in the pathogenesis of aGVHD. The change of CTLA4 showed that its expression level in aGVHD was different in stages. We speculate that the expression level increases rapidly in the period of T cell activation and down-regulates in the effect stage. These suggest that CTLA4 is suitable as an early biomarker of aGVHD and less suitable as a diagnostic marker, and that CD28 may have the potential to serve as an earlier marker of aGVHD.

CD25 is one of the markers of T-cell activation (33). CD25 plays an important role in promoting T cell activation, proliferation, and differentiation into effector T cells. When T cells activated, the expression of CD25 on T cells increases. Several studies have confirmed that CD25 in the serum of patients with aGVHD increases (19–25). Our previous studies also confirmed that the level of CD25+T cells in peripheral blood was elevated (34). In the aGVHD group, CD25 mRNA showed an upward trend from day +7 to day +14 post-transplantation (P=0.09), and a downward trend from day +14 to day +21 (P=0.057). Our results show changes in transcript levels of CD25 in the early stages of aGVHD.

The results of this study showed that there was no difference in the transcription level of CD28 between the two groups with aGVHD and non-aGVHD on day +7, +14 and +21 post-transplantation, and that there were differences in CD25 and CTLA4 transcript levels day +14, but there were no differences on day +7 and +21. Because CTLA4 plays an inhibitory role in T cell activation, it is generally believed that CTLA4 plays a protective role in the pathological process of aGVHD, and low levels of CTLA4 predict the occurrence and development of aGVHD. It has also been suggested that due to the presence of soluble CTLA4 in the peripheral circulation, the binding of B7 (CD80/86) to both CD28 and membrane-bound CTLA-4 can be blocked, attenuating the down-regulatory effect on T cells (35). Ramzi M and other scholars have shown that the expression of CD28 in GVHD patients was higher than that in non-GVHD patients, while the expression of CTLA4 in GVHD patients was slightly lower than that in non-GVHD patients (36). It has also been demonstrated that CTLA4 levels were higher in patients with grade I-II aGVHD than in patients with grade III-IV aGVHD both peri- and post- treatment (37). However, some research results have not come to the same conclusion. Tanaka J study concluded that CD28 and CTLA4 play an important role in inducing strong allogeneic responses and that CD28 and CTLA-4 mRNA expression is elevated in patients with severe aGVHD (38). Our results also showed that the transcriptional level of CTLA4 was significantly increased in aGVHD group. The reason for the inconsistent results, we believe, is related to the difference in CTLA-4 stage expression in aGVHD, which was caused by the different time points of specimen collection.

By ROC analysis, the AUCs of CD25 and CTLA4 mRNA on day +14 post-transplantation to predict the occurrence aGVHD were 0.7267 (73.30%,66.52%) and 0.7167 (66.67%,72.00%), with cut-off values of 1.032 and 2.734, respectively. The AUC of the combined CD25 and CTLA4 mRNA to predict the occurrence of aGVHD was 0.7613 (96.67%,50.00%), and the AUC of the combined prediction was slightly increased. The AUC of CTLA4 mRNA on day +7 to predict grade II-IV aGVHD was 0.8287(88.89%,75.00%), and the cut-off value was 1.240. The AUCs of CTLA4 mRNA on day +7 and CD25 mRNA on day +14 to predict gastrointestinal aGVHD were 0.7593 (81.25%,70.31%) and 0.6602 (75.00%,57.81%), and the Cut-off values were 2.687 and 1.240, respectively. The results showed that CTLA4 mRNA had the potential of predicting the occurrence of aGVHD, grade II-IV aGVHD and gastrointestinal aGVHD, and CD25 mRNA had the ability of early prediction of the occurrence of aGVHD and gastrointestinal aGVHD.

While the frequency of T cell and T cell subsets in the PBMC samples were not determined, CD25 and CTLA4 mRNA expressions in PBMC were increased in aGVHD patients compared to those in non-GVHDpatients on day 7 and 14 post transcriptional. Given the poor cellularity before engraftment, qPCR on peripheral blood PBMC is a feasible and convenient method to predict aGVHD and consider early intervention. Expression levels of CD25, CD28, and CTLA-4 vary among lymphocyte subtypes, with higher CD25 and CTLA-4 expression on CD4+ regulatory T cells (Tregs). The proportion of lymphocyte subpopulations might influence results. Unfortunately, we did not measure lymphocyte subpopulation counts at each time point, preventing an assessment of their impact. The cellular sources of those increased RNA expression such as Tregs and exhausted T cells will be studied in a future study. This limitation underscores the need for further exploration in future studies.

## 5 Conclusions

The relative expression of CD25 and CTLA4 mRNA were increased in aGVHD patients, which had early predictive value for the occurrence of aGVHD, grade II-IV aGVHD and gastrointestinal aGVHD, and can be used as early biomarkers for aGVHD.

## Data availability statement

The original contributions presented in the study are included in the article/supplementary materials, further inquiries can be directed to the corresponding author/s.

## Ethics statement

The studies involving humans were approved by the Medical Ethics Committee of First Affiliated Hospital of Guangxi Medical University. The studies were conducted in accordance with the local legislation and institutional requirements. Written informed consent for participation in this study was provided by the participants’ legal guardians/next of kin. Written informed consent was obtained from the individual(s), and minor(s)’ legal guardian/next of kin, for the publication of any potentially identifiable images or data included in this article.

## Author contributions

KH: Conceptualization, Data curation, Formal analysis, Investigation, Methodology, Project administration, Resources, Visualization, Writing – original draft, Writing – review & editing. MY: Data curation, Investigation, Methodology, Resources, Visualization, Writing – original draft, Writing – review & editing. YZ: Data curation, Investigation, Writing – review & editing. YC: Data curation, Investigation, Writing – review & editing. GP: Data curation, Investigation, Writing – review & editing. JZ: Data curation, Investigation, Writing – review & editing. YL: Data curation, Investigation, Writing – review & editing. JL: Conceptualization, Funding acquisition, Resources, Supervision, Writing – review & editing.
